# Efficacy of a multicomponent singing intervention on communication and psychosocial functioning in chronic aphasia: a randomized controlled crossover trial

**DOI:** 10.1093/braincomms/fcac337

**Published:** 2022-12-27

**Authors:** Sini-Tuuli Siponkoski, Anni Pitkäniemi, Sari Laitinen, Essi-Reetta Särkämö, Emmi Pentikäinen, Heidi Eloranta, Leena Tuomiranta, Susanna Melkas, Gottfried Schlaug, Aleksi J Sihvonen, Teppo Särkämö

**Affiliations:** Cognitive Brain Research Unit, Department of Psychology and Logopedics, University of Helsinki, 00014 Helsingin yliopisto, Helsinki, Finland; Centre of Excellence in Music, Mind, Body and Brain, University of Helsinki, 00014 Helsingin yliopisto, Helsinki, Finland; Cognitive Brain Research Unit, Department of Psychology and Logopedics, University of Helsinki, 00014 Helsingin yliopisto, Helsinki, Finland; Centre of Excellence in Music, Mind, Body and Brain, University of Helsinki, 00014 Helsingin yliopisto, Helsinki, Finland; Centre of Excellence in Music, Mind, Body and Brain, University of Helsinki, 00014 Helsingin yliopisto, Helsinki, Finland; Espoo Hospital, 00029 HUS, Espoo, Finland; Private choir conductor, 01300 Vantaa, Finland; Cognitive Brain Research Unit, Department of Psychology and Logopedics, University of Helsinki, 00014 Helsingin yliopisto, Helsinki, Finland; Centre of Excellence in Music, Mind, Body and Brain, University of Helsinki, 00014 Helsingin yliopisto, Helsinki, Finland; Helsinki-Uusimaa Stroke Association, 00610 Helsinki, Finland; Department of Psychology and Logopedics, University of Helsinki, 00014 Helsingin yliopisto, Helsinki, Finland; Department of Neurology, University of Helsinki and Helsinki University Central Hospital, 00029 HUS, Helsinki, Finland; Department of Neurology, UMass Medical School, Springfield & Department of Biomedical Engineering and Institute of Applied Life Sciences, UMass Amherst, Amherst, MA 01003, USA; Cognitive Brain Research Unit, Department of Psychology and Logopedics, University of Helsinki, 00014 Helsingin yliopisto, Helsinki, Finland; Centre of Excellence in Music, Mind, Body and Brain, University of Helsinki, 00014 Helsingin yliopisto, Helsinki, Finland; Department of Neurology, University of Helsinki and Helsinki University Central Hospital, 00029 HUS, Helsinki, Finland; School of Health and Rehabilitation Sciences, Queensland Aphasia Research Centre and UQ Centre for Clinical Research, The University of Queensland, QLD 4029, Brisbane, Australia; Cognitive Brain Research Unit, Department of Psychology and Logopedics, University of Helsinki, 00014 Helsingin yliopisto, Helsinki, Finland; Centre of Excellence in Music, Mind, Body and Brain, University of Helsinki, 00014 Helsingin yliopisto, Helsinki, Finland

**Keywords:** singing, rehabilitation, aphasia, communication, speech production

## Abstract

The ability to produce words through singing can be preserved in severe aphasia, but the benefits of group-based singing rehabilitation in aphasia are largely unknown. Our aim was to determine the efficacy of a multicomponent singing intervention on communication and speech production, emotional-social functioning and caregiver well-being in aphasia. Fifty-four patients with acquired brain injury and chronic aphasia and their family caregivers (*n* = 43) were recruited. Using a crossover randomized controlled trial design, participants were randomized to two groups who received a 4-month singing intervention either during the first or second half of the study in addition to standard care. The intervention comprised weekly group-based training (including choir singing and group-level melodic intonation therapy) and tablet-assisted singing training at home. At baseline, 5- and 9-month stages, patients were assessed with tests and questionnaires on communication and speech production, mood, social functioning, and quality of life and family caregivers with questionnaires on caregiver burden. All participants who participated in the baseline measurement (*n* = 50) were included in linear mixed model analyses. Compared with standard care, the singing intervention improved everyday communication and responsive speech production from baseline to 5-month stage, and these changes were sustained also longitudinally (baseline to 9-month stage). Additionally, the intervention enhanced patients’ social participation and reduced caregiver burden. This study provides novel evidence that group-based multicomponent singing training can enhance communication and spoken language production in chronic aphasia as well as improve psychosocial wellbeing in patients and caregivers.

https://www.clinicaltrials.gov, Unique identifier: NCT03501797.

## Teaser

Siponkoski *et al*. report that group-based multicomponent singing training can enhance communication and spoken language production in chronic aphasia as well as improve psychosocial wellbeing in patients and caregivers. The findings demonstrate that singing provides a versatile, motivating, scalable, and potentially cost-effective approach to aphasia rehabilitation.

## Graphical Abstract

**Graphical abstract:**
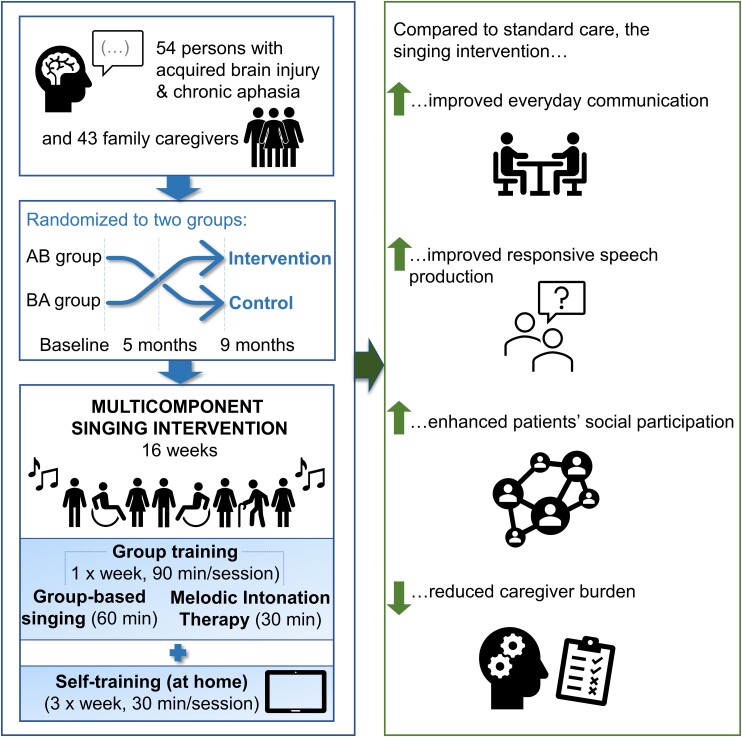

## Introduction

Aphasia is a highly debilitating condition that impairs communication abilities, causing social isolation and decreasing emotional wellbeing.^1^ The leading cause of aphasia is stroke: about 40% of stroke survivors experience aphasia, and in half of them, the communication impairment persists after 1 year post-stroke.^2^ Aphasia reduces quality of life (QoL) more than other stroke-induced deficits^3^ or many severe chronic illnesses, including cancer and Alzheimer’s disease.^4^ Considering the high prevalence of stroke and the sustained burden caused by aphasia on the survivors, their families and the entire society, there is a pressing need for new effective, easily applicable and scalable treatments that target both the communicative and psychosocial needs of persons with aphasia (PWAs). This is particularly true at the chronic stage when PWAs typically no longer receive active treatment, even though it can be effective also at this stage,^5,6^ and often experience social exclusion^7^ as well as for their family caregivers (FCs), who face high burden and are at risk of developing depression and anxiety.^8^

Music is a versatile and effective rehabilitation tool, which can support motor, cognitive and emotional recovery after stroke^9–11^ but which has thus far not been translated effectively to clinical practice in treating chronic post-stroke aphasia. In aphasia, the ability to vocalize through singing is often preserved,^12^ and singing can help the motor production of words for example by slowing down the rate of vocal production, entraining it to the musical rhythm and increasing the connectedness between syllables/words.^13^ Various singing-based aphasia rehabilitation methods have been developed, including the melodic intonation therapy (MIT) where the production of formulaic speech phrases is trained together with a therapist using melodic intoning (singing) and rhythm (hand tapping), following a protocol that progresses from singing to the production of speech with more natural prosody.^14,15^ MIT has shown promise in enhancing functional communication and expressive language in aphasia,^16,17^ although larger randomized controlled trials (RCTs) are still needed to provide definite evidence on its clinical efficacy.^18^ Notably, these methods have mostly been applied in individual-level rehabilitation, which not only requires extensive personnel resources but also overlooks the emotional, communicative and social-interactive power of singing when done in a group.

Group-based singing is a viable and multifaceted approach for aphasia rehabilitation, because it combines verbal production with expressive music making, enjoyable social interaction and peer support. The emotional, social and cognitive benefits of choir singing have been recognized in healthy older adults,^19,20^ among whom it has become a very popular activity. In PWAs, choir singing has thus far been explored in three small-scale pilot studies. In a within-subject study of nine PWAs, Tamplin *et al*.^21^ reported a trend towards reduction of psychological distress after a 20-week community choir intervention (2 h sessions once a week) comprising singing familiar songs, vocal exercises and socialization and led by a music therapist and assisted by volunteers. Qualitatively, positive effects of the choir intervention on confidence, peer support, mood, motivation and communication were observed in interviews of PWAs.^21^ In a three-arm pilot study of 15 PWAs, Zumbansen *et al*.^22^ found no significant improvement in functional communication, expressive language, mood or QoL after a 26-week choir intervention (2 h sessions once a week) compared with a control intervention (drama) or standard care but reported a correlation between attendance to social activities and improvement in functional communication. Recently, Tarrant *et al*.^23^ reported a two-arm feasibility study of 36 PWAs in which a 10-week group singing intervention (90 min sessions once a week) led by a community musician and assisted by a PWA volunteer was found to be acceptable and safe for PWAs. These studies provide proof of concept that group singing is a feasible intervention for PWAs and suggest that its clinical efficacy should be explored in a larger clinical trial.^24^

In summary, previous studies suggest that MIT and choir singing are promising tools for enhancing communicative and psychosocial functioning, respectively, but their synergistic combination within the same group intervention protocol has never been explored. Likewise, previous studies have not considered the role of FC participation and added home training in PWA singing interventions. We developed a new multicomponent singing intervention for PWAs, which (i) combines choir singing and MIT adapted for group-level training to target both communicative and psychosocial outcomes, (ii) is aimed both for PWAs and their FCs to support their interaction and provide enjoyable joint activity and peer support to both, to reduce caregiver burden and to facilitate the translation of practiced functions and skills to the everyday life of the PWAs and (iii) includes tablet-assisted singing training at home to increase the intensity of the intervention and enable the learning of the choir songs. The aims of the multicomponent intervention were to improve communication and spoken language production and emotional, social and functional outcomes in PWAs and psychological well-being in FCs.

In order to determine the clinical efficacy of the intervention, we performed a single-blind crossover RCT in PWAs (*N* = 54) and their FCs (*n* = 43) comparing the multicomponent singing intervention to standard care from baseline (T1) to 5-month (T2) and 9-month (T3) follow-up stages. We hypothesized that compared with standard care, the multicomponent singing intervention would enhance communication skills in the PWAs as the primary outcome (from T1 to T2) as well as lead to improvements in spoken language production, verbal memory, mood and QoL in the PWAs and in caregiver burden in the FCs as secondary outcomes.

## Materials and methods

### Participants and study design

Fifty-four PWAs with a history of cerebrovascular accident (*n* = 53) or traumatic brain injury (*n* = 1) leading to aphasia and their FCs (*n* = 43) were successfully recruited from the Helsinki region during 2017–19 through patient organizations (Helsinki-Uusimaa Stroke Association, Finnish Brain Association) and clinical speech therapists. The FCs were spouses (*n* = 22), children (*n* = 8), siblings (*n* = 2), parents (*n* = 4) and others (*n* = 7). The recruiting psychologists interviewed all PWAs interested in the study for evaluating eligibility. The inclusion criteria were (i) age ≥ 18; (ii) Finnish-speaking; (iii) time since stroke/injury > 6 months; (iv) at least mild aphasia [Boston Diagnostic Aphasia Examination (BDAE) Aphasia Severity Rating Scale^25^ score ≤4 (preliminary assessment based on recruitment interview)]; (v) no subjective hearing deficit; (vi) cognitive ability to give an informed consent; (vii) no neurological/psychiatric co-morbidity or substance abuse and (viii) ability to produce vocal sound through singing/humming. The study was approved by the Ethics committee of the Helsinki-Uusimaa Hospital District, and written informed consents were obtained from all PWAs and FCs.

**Figure 1:**
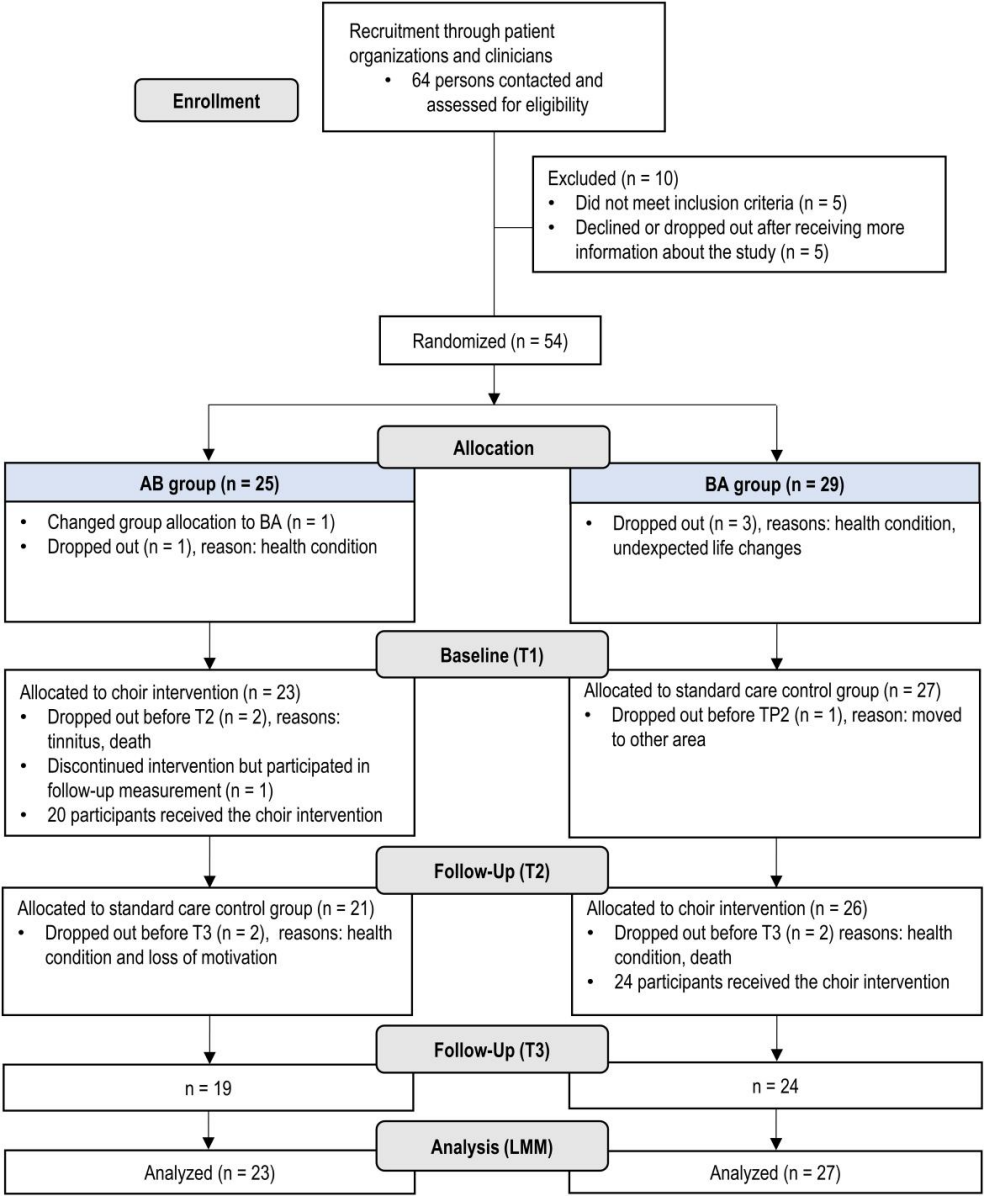
**Flowchart outlining the design and progress of the trial**. LMM, linear mixed effects model; T1, timepoint 1 (baseline); T2, timepoint 2 (5-month); T3, timepoint 3 (9-month).

The study was implemented using a crossover RCT in order to enable access to treatment for all participants and maximize the data on intervention experiences. The intervention and the study are reported according to the TIDieR^26^ and CONSORT^27^ guidelines. Fig. 1 shows a flowchart of the study design. In two data collection waves (2018: *n* = 33, 2019: *n* = 21), the PWA participants were randomly assigned to two groups (AB/BA, A = intervention, B = control) stratified for aphasia severity (preliminary BDAE severity level), FC’s participation in group sessions, sex, age and time since stroke/injury. The randomization was performed for matched pairs using an online random number generator (https://www.random.org) by a researcher not involved in data collection.

The outcome measures, including neuropsychological and language tests and questionnaires, were performed at baseline (T1), 5-month mid-point (T2) and 9-month endpoint (T3). Additionally, MRI and EEG data were gathered from a subgroup of PWAs, in addition to which quantitative and qualitative feedback was collected from PWAs and FCs after the intervention period; these results will be reported separately. AB received the singing intervention during the first 16-week period (T1 and T2) and BA received it during the last 16-week period (T2 and T3). Throughout the trial, both groups received standard care, comprising the standard speech therapy, neuropsychological rehabilitation and physical/occupational therapy provided in public health care.

The drop-out rates and reasons are presented in Fig. 1. Some PWAs discontinued the study due to health problems, and there were two deaths not attributed to the study. One PWA reported that group singing triggered tinnitus, no other adverse effects or harms were reported by the PWAs or FCs.

Additional 23 PWAs were recruited from Southwest Finland for a third study wave and underwent T1 in January–February 2020, but the trial was stopped due to the COVID-19 pandemic in March 2020 and their data had to be excluded from the present study. The sample size was determined based on previous music-intervention studies and practical possibilities in finding participants.

### Intervention

The 16-week multicomponent singing intervention was a combination of group training (1 session/week, 1.5 h/session, total 24 h) held at a local aphasia centre (https://www.afasiakeskus.fi/) and home training (target: 3 sessions/week, 30 min/session, total 24 h). Implemented by a two-person team (choir conductor and music therapist, authors E.-R.S. and S.L.), the intervention was held separately for 4 groups of participants (2 AB groups and 2 BA groups; 10–14 PWAs and 6–10 FCs per group). Thirty-two (AB = 14, BA = 18) FCs joined the group sessions; the rest participated only as informants. To enhance treatment conformity, the intervention was administered to all groups by the same two-person team, in accordance with *a priori* fixed protocol described below.

#### Group training

Group training sessions comprised 60 min of singing training for PWAs and FCs and 30 min of group-based MIT for PWAs. The singing training was implemented in an encouraging group environment and consisted of breathing and vocal exercises and voice warm-ups (20 min/session), aimed at strengthening voice intensity and syllable-level vocal production, and group singing with choral elements (40 min/session). The group training was implemented in a spacious lobby area, with easy wheel chair access and chairs arranged in a semi-circle around the choir conductor and a screen to which song lyrics were projected during training. The song repertoire (10 songs) mainly consisted of highly familiar Finnish popular and folk songs (for facilitating word retrieval and recall) and a few novel songs (for learning new verbal material), accompanied with piano during the training. The songs were especially arranged for PWAs and FCs, with keys selected for novice singers and tempos slowed down to ease word production, and included also polyphonic arrangements where PWAs sang melody and FCs sang second melody. After each group training session, there was a short voluntary social get together with coffee/tea, which most participants took part in. Each group rehearsed to perform the songs for a small concert held for family and friends in the last session, bringing a goal-oriented element to the training.

Adapted from the original MIT,^14,15^ the group-based MIT (30 min/session) comprised singing-based training of formulaic speech phrases, incorporating the key elements of MIT [simple melodic structure, simultaneous tapping with the non-paretic (left) hand, stepwise progression from modelling and unison production to repetition]. In our adapted MIT protocol, the training followed a simple five-step cycle for each phrase: (i) thinking (mental preparation), (ii) sung production with melodic intonation, (iii) sung production with melodic intonation and rhythmic pacing (hand tapping for stressed syllables), (iv) spoken production with rhythmic pacing and (v) natural spoken production (without pacing). In this cycle, the therapist first provides a model for each step, which the PWA then performs. Augmentative and Alternative Communication pictures depicting the phrases were also used as visual aids during the MIT training.

The group-based MIT was used in 14 sessions (excluding first and last session). In the first 7 sessions, the PWAs rehearsed a set of 10 formulaic phrases with the music therapist using the MIT protocol, while the FCs trained the second melody of polyphonic songs with the choir conductor in another room. In the latter seven sessions, once the PWAs had internalized the MIT protocol, the FCs joined the PWAs and they trained together using MIT in reciprocal dialogue situations themed around everyday life (e.g. having dinner with guests, cleaning the house). For this, the participants were split to two groups with lead singers. In the first group, the lead singer produced a melodically intoned phrase (e.g. ‘Welcome!’) which the first group then repeated. After this, the lead singer of the second group produced a dialogic response to the first phrase (e.g. ‘Thank you!’), correspondingly repeated by the second group. Using this cycle, the groups had short conversations, aimed at translating the MIT protocol to daily life.

#### Home training

Singing in a choir usually entails self-training of the song material at home. To facilitate the learning of the song material and to increase the intensity of the training, a tablet-based training application called Singalonger was developed together with a Finnish company (Outloud). Singalonger was used on a tablet computer (Samsung Galaxy Tab 4) and a headset microphone (Logitech H151), provided to each participant. The application included all the songs that were in the choir repertoire and had three options for training aids that the participants could select when singing along to each song: (i) an instrumental auditory model or a sung (female/male) auditory model, (ii) karaoke-type printed lyrics running on the screen (in time with melody) and (iii) a video showing the mouth movements of the model female/male singer (helping to imitate the movements). Singalonger automatically recorded the singing of the participant and analysed the pitch and length of each sung note, which enabled providing online feedback (star-rating) to encourage and motivate training. The patients were trained in using the tablet and application in the first group session and then and instructed to train the song material by themselves using Singalonger three times a week (30 min/session) for the following 16 weeks. The participants also received easy-to-follow pictorial instructions and had technical support available throughout the intervention on how to use the tablet and application.

### Outcome measures

The neuropsychological and language assessments were conducted by trained psychologists (authors S.-T.S., A.P. and E.P.) at the Cognitive Brain Research Unit. All spoken language production tasks were recorded. The questionnaires were sent prior to the testing session to PWAs and FCs and were returned to the psychologist. FCs were instructed to help the PWA only in reading the questions without giving guidance in answering. The researchers conducting the assessments and analysing the data were blinded to the group allocation of the participants until the final statistical analysis when the AB/BA groups were compared with each other.

**Table 1:** Baseline clinical and demographic background information of the PWAs

	All (*n* = 50)	AB (*n* = 23)	BA (*n* = 27)	Difference between groups (*P* value)
**Demographic information**				
Age	64.0 (12.3)	63.5 (10.3)	64.5 (14.0)	0.787 (*t*)
Sex (female/male)	28/22	11/12	17/10	0.283 (χ^2^)
Handedness (right/left)	42/8	21/2	21/6	0.261 (*F*)
Education level^a^	2.9 (1.4)	3.0 (1.4)	2.9 (1.4)	0.965 (*t*)
**Clinical information**				
Aetiology of injury (ischaemic/haemorrhagic/both/TBI)	28/16/3/1	14/6/2/0	14/10/1/1	0.641 (*F*)
Time since injury (months)	73.3 (68.4)	76.0 (69.5)	71.0 (68.7)	0.789 (*t*)
Aphasia severity (mild/moderate or severe)^b^	34/16	14/9	20/7	0.318 (χ^2^)
**Musical background**				
Choir singing years	3.1 (9.2)	4.0 (11.4)	2.0 (5.2)	1.000 (U)
Singing lessons years	0.5 (2.8)	0.1 (0.5)	0.8 (3.7)	0.671 (U)
Instrument lessons years	1.4 (3.2)	0.6 (1.4)	2.3 (4.2)	0.201 (U)

Data are mean (SD) unless otherwise stated.

*t*, independent-samples *t*-test; *F*, Fisher’s exact test; *U*, Mann–Whitney U-test; χ^2^, Chi-squared test.

^a^ Education level according to the UNESCO International Standard Classification of Education: range 1 (primary education) to 6 (doctoral or equivalent level).

^b^ Aphasia severity based on the WAB AQ rate, Score 0–50 = severe, Score 51–100 = mild/moderate.

Our primary outcome was change in communication ability from T1 to T2. Secondary outcomes were change in communication ability from T1 to T3 and changes in spoken language production and verbal skills and emotional, social and functional outcome from T1 to T2 and T1 to T3.

#### Communication ability

Communication ability was measured with the Communicative Activity Log (CAL)^28^ and the Communication subscale of the Stroke Impact Scale 3.0 (SIS),^29^ which were both administered to PWAs (self-report) and FCs (informant-report). These measures were chosen to capture changes in daily communication induced by the intervention in an ecologically valid way. In order to gain a comprehensive picture of communication skills in the sample, independent of aphasia severity level, and to pool measures to reduce the amount of analysis and the risk of type I errors,^30^ we calculated a common communication index by averaging the percentage scores (score/total × 100) of the PWAs and FCs in the CAL communication total score and SIS communication subscale score (reversed to match the CAL).

#### Spoken language production and verbal skills

Spontaneous speech was assessed with the Spontaneous speech index of the Western Aphasia Battery (WAB).^31^ The Repetition and Naming indices of WAB were used to assess more automatic and stimulus-dependent spoken language production. They were averaged together to form a Responsive speech index, similar to previous studies.^32^ At baseline, we also calculated the WAB Aphasia Quotient (AQ),^31^ indicating the overall severity level of the aphasia, from the Spontaneous speech, Repetition, Naming and Comprehension (estimated based on the Sequential commands subtest) indices. Additionally, we evaluated motor speech production (apraxia of speech) using the articulatory agility subtest of BDAE^25^ and a verbal memory index from the average of the percentage scores (score/total × 100) of the Logical Memory and Word Lists subtests of Wechsler Memory Scale III,^33^ and the Finnish KAT verbal working memory task.^34^ Parallel versions of the memory tasks were used for the T1-T2-T3 measurements, and their orders were randomized and balanced between AB/BA groups.

#### Emotional, social and functional outcomes

Functional impairment of PWAs was assessed with four SIS subscales: physical functioning (average of ADL/IADL, strength, hand function, and mobility), emotion, memory and thinking, and participation and role function. The percentage scores of the PWAs and FCs were averaged together. PWAs’ self-evaluated mood (depression) and social support were measured using the percentage scores of the Center for Epidemiological Studies-Depression (CES-D)^35^ and Social Provision Scale (SPS),^36^ respectively. The General Health Questionnaire (GHQ-12)^37^ and Zarit Burden Interview (ZBI-22)^38^ were administered to the FCs and their average percentage score was used as an index of caregiver burden.

### Statistical analysis

The statistical analyses were conducted using IBM SPSS Statistics 26.

#### Intention-to-treat analysis

Main analyses were conducted using linear mixed effects model (LMM) on the whole sample of participants who participated in the first measurement (*n* = 50). This approach utilizes all available data according to the intention-to-treat (ITT) principle.^39^ Time × Group interactions were analysed between T1 and T2 using repeated measures analysis (restricted maximum likelihood method), in which Group and Time were included in the model as fixed effects and within-subject variation as a random effect. Compound symmetry was selected as the covariance structure based on Schwarz’s Bayesian Criterion. For measures yielding significant effects in LMM, the long-term effects were further investigated within both AB and BA groups over T1–T3. Direct comparisons between groups were not conducted between T2 and T3 due to the possible carry-over effect in the AB group. Effect sizes were approximated using the repeated measures ANOVA, because the LMM procedure does not produce effect sizes in SPSS, and the chosen LMM model and covariance structure were very similar with traditional ANOVA.

#### Per-protocol analysis

To evaluate the sensitivity of our results in a dataset of subjects who adhered to study protocol and participated in all measurement points (between T1 and T2: AB = 20, BA = 26), we performed a per-protocol (PP) analysis for the significant measures from the LMM analysis using repeated measures ANOVA. One PWA who participated in T1 and T2 measurements, but dropped out of the intervention, was excluded from the analysis due to protocol violation. Additionally, pre- and post-intervention (AB: T1 and T2, BA: T2 and T3) scores were compared for the significant measures using paired *t*-tests.

## Results

Clinical and demographic characteristics of the PWAs are presented in Table 1, and their adherence to the intervention and amount of other rehabilitation (standard care) received during the study period in Table 2. The AB and BA groups did not differ significantly in any of these variables. Also, the demographic characteristics of the FCs (*n* = 43, 30 females, mean age 61.4 years) did not differ between AB/BA.

**Table 2:** Amount of standard rehabilitation and group and home training

	All (*n* = 50)	AB (*n* = 23)	BA (*n* = 27)	Difference between groups (*P* value)
**T1–T3**				
Home training^a^	11.9 (9.8)	13.7 (11.0)	10.4 (8.6)	0.321 (U)
Group training (attendance rate)	90.1% (14.0)	89.0% (18.0)	92.4% (9.4)	0.426 (*t*)
Speech therapy	9.75 (12.4)	7.8 (12.4)	11.3 (12.5)	0.232 (U)
Physical therapy	8.9 (15.0)	9.2 (16.2)	8.8 (14.4)	0.848 (U)
Occupational therapy	1.8 (4.3)	1.8 (5.2)	1.7 (3.6)	0.740 (U)
Neuropsychological rehabilitation	0.2 (0.8)	0.1 (0.4)	0.3 (1.0)	0.663 (U)
**T1–T2**				
Speech therapy	6.5 (8.6)	4.2 (2.6)	7.3 (7.5)	0.332 (U)
Physical therapy	6.2 (9.0)	3.7 (5.4)	5.1 (7.3)	0.778 (U)
Occupational therapy	1.4 (3.7)	0.1 (4.1)	0.9 (1.9)	0.751 (U)
Neuropsychological rehabilitation	0.4 (1.6)	0.0 (0.0)	0.3 (1.0)	0.724 (U)
**T2–T3**				
Speech therapy	4.1 (5.8)	4.4 (6.5)	5.1 (5.8)	0.288 (U)
Physical therapy	4.1 (7.4)	3.8 (6.1)	4.2 (8.4)	0.566 (U)
Occupational therapy	0.5 (2.0)	0.1 (0.2)	1.1 (2.8)	0.386 (U)
Neuropsychological rehabilitation	0.1 (0.2)	0.1 (0.4)	1.0 (0.0)	0.258 (U)

Data are mean (SD) in hours unless otherwise stated.

^a^ Based on Singalonger log files. Abbreviations: U = Mann–Whitney U-test; *t* = independent-samples *t*-test; T1, timepoint 1 (baseline); T2, timepoint 2 (5-month); T3, timepoint 3 (9-month).

### Communication and spoken language production outcome

**Figure 2:**
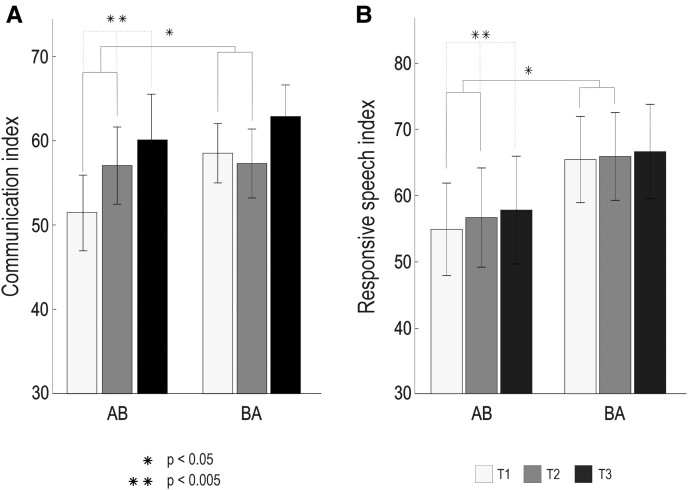
**Communication and speech production results from the LMM analysis (*N* = 50)**. (**A**) Communication index (*F* = 7.082, *P* = 0.011). (**B**) Responsive speech index (*F* = 4.100, *P* = 0.049). AB received the intervention from T1 to T2, and BA received the intervention from T2 to T3. The statistical method used in this study was the LMM analysis. The bar plots (mean—SEM) show changes in test scores over the three time-points (T1–T3) presented group-wise (AB/BA). Significant Time × Group interactions are shown with solid lines and significant within-group Time main effects are shown with dashed lines. LMM, linear mixed effects model; SEM, standard error of the mean; T1, timepoint 1 (baseline); T2 timepoint 2 (5-month); T3 timepoint 3 (9-month).

The ITT results from communication and spoken language production Time × Group LMM analysis (T1 and T2) are presented in Table 3 and Fig. 2. There were significant improvements in the AB group compared with the BA group between T1 and T2 in the Communication index [*F*(1,45) = 7.08, *P* = 0.011, η*p*^2^ = 0.140] and in the Responsive speech index [*F*(1,45) = 4.10, *P* = 0.049, η*p*^2^ = 0.084]. No significant effects were observed in the other measures.

**Table 3:** Communication and spoken language outcome results from LMM analysis

Measure	Group	T1 mean (SD)	T2 mean (SD)	T3 mean (SD)	Observations T1/T2/T3	Baseline diff.	Δ T1–T2 (*F* value)	Δ T1–T2 (*P* value)
**Communication**								
**Communication index** (percentage)^a^	AB	51.6 (21.6)	57.1 (21.0)	60.2 (22.4)	50/47/40	0.219	7.082	**0**.**011**
	BA	58.6 (18.4)	57.4 (20.9)	63.0 (18.0)				
**Spoken language production**								
**Spontaneous speech index** (percentage)^a^	AB	60.7 (32.8)	62.6 (32.7)	63.4 (32.4)	50/47/42	0.223	0.264	0.610
	BA	70.2 (30.0)	71.5 (30.3)	72.4 (29.8)				
**Responsive speech index** (percentage)^a^	AB	55.0 (33.5)	56.7 (34.3)	57.9 (34.5)	50/47/40	0.276	4.100	**0**.**049**
	BA	65.5 (33.8)	65.9 (33.7)	66.7 (33.4)				
**Articulatory agility** (percentage)^a^	AB	49.0 (29.5)	49.7 (31.5)	47.4 (31.2)	49/47/42	0.442	0.308	0.582
	BA	55.6 (33.8)	54.1 (33.5)	58.7 (34.5)				
**Verbal memory**								
**Verbal memory index** (percentage)^a^	AB	27.1 (15.6)	26.6 (16.6)	29.4 (16.5)	50/47/42	0.370	2.022	0.191
	BA	30.1 (15.5)	31.9 (16.0)	33.2 (18.7)				

^a^ Higher score indicates better outcome.

Values in bold indicate statistically significant results. T1, timepoint 1 (baseline); T2, timepoint 2 (5-month); T3, timepoint 3 (9-month).

Within-group longitudinal analyses showed that the Time main effect (T1–T3) in the AB group was significant in the Communication index [*F*(2,37) = 6.44, *P* = 0.004, η*p*^2^ = 0.308] and in the Responsive speech index [*F*(2,37) = 6.87, *P* = 0.003, η*p*^2^ = 0.222]. *Post hoc* pairwise comparisons indicated that in both the Communication and the Responsive speech index, the outcome improved between T1 and T2 (*P* = 0.013 and *P* = 0.001) and between T1 and T3 (*P* = 0.002 and *P* = 0.009) but did not change between T2 and T3 (*P* = 0.337 and *P* = 0.608) in the AB group, suggesting that the gains from the intervention were maintained at the longitudinal follow-up. These comparisons survived after false discovery rate (FDR)-correction. Within the BA group, there were no significant Time (T1–T3) main effects in either index [Communication: *F*(2,47) = 2.02, *P* = 0.144, η*p*^2^ = 0.079; Responsive speech: *F*(2,46) = 1.567, *P* = 0.220, η*p*^2^ = 0.068], but the changes were in a positive direction.

### Functional, emotional and social outcome

**Figure 3:**
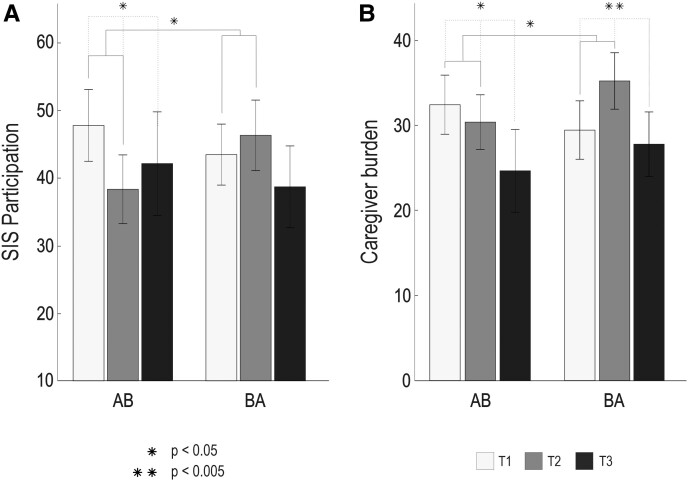
**Functional outcome and caregiver burden results from the LMM analysis (*N* = 50)**. (**A**) SIS participation and role function (*F* = 6.440, *P* = 0.015). (**B**) Caregiver burden as measured by GHQ-12 and ZBI-22 (*F* = 6.765, *P* = 0.014). AB received the intervention from T1 to T2, and BA received the intervention from T2 to T3. The statistical method used in this study was the LMM analysis. The bar plots (mean—SEM) show changes in test scores over the three time-points (T1–T3) presented group-wise (AB/BA). Significant Time × Group interactions are shown with solid lines. Time main effects are shown with dashed lines. GHQ-12, General Health Questionnaire 12; LMM, linear mixed effects model; SEM, standard error of the mean; SIS, Stroke Impact Scale; T1, timepoint 1 (baseline); T2, timepoint 2 (5-month); T3, timepoint 3 (9-month); ZBI-22, Zarit Burden Interview 22.

The ITT results from functional, emotional and social outcome measures are shown in Table 4 and Fig. 3. In the Time × Group LMM analysis, a significant improvement in the AB versus BA group between T1 and T2 was found in the SIS participation and role function subscale [*F*(1,43) = 6.44, *P* = 0.015, η*p*^2^ = 0.139] and in the Caregiver burden index [*F*(1,40) = 6.77, *P* = 0.014, η*p*^2^ = 0.177]. No significant effects were found in the other measures.

**Table 4:** Functional, social, mood and cognitive outcomes analysed using LMM

Measure	Group	T1 mean (SD)	T2 mean (SD)	T3 mean (SD)	Observations T1/T2/T3	Baseline diff.	Δ T1–T2 (*F* value)	Δ T1–T2 (*P* value)
**Functional impairment**								
**SIS physical functioning** (percentage)	AB	37.7 (23.7)	37.2 (25.0)	35.5 (26.1)	48/47/40	0.613	0.223	0.639
	BA	34.1 (23.7)	34.9 (25.2)	32.6 (22.6)				
**SIS emotion** (percentage)	AB	29.3 (17.2)	27.1 (14.2)	23.9 (19.3)	50/47/40	0.703	0.799	0.376
	BA	27.6 (10.3)	27.3 (10.4)	23.5 (12.0)				
**SIS memory and thinking** (percentage)	AB	30.1 (19.8)	27.6 (20.4)	25.7 (20.9)	50/47/40	0.386	1.312	0.258
	BA	25.5 (17.5)	25.8 (19.9)	23.2 (17.5)				
**SIS participation and role function** (percentage)	AB	47.7 (24.9)	38.3 (23.3)	42.1 (31.5)	47/47/40	0.537	6.440	**0**.**015**
	BA	43.4 (22.5)	46.3 (26.5)	38.7 (29.0)				
**Mood (depression)**								
**CES-D total score** (percentage)	AB	27.8 (13.1)	25.2 (14.7)	22.14 (12.6)	50/47/40	0.613	0.136	0.715
	BA	29.5 (11.2)	28.0 (10.6)	29.9 (12.4)				
**Social support**								
**SPS total score** (percentage)^a^	AB	82.21(12.6)	80.1 (12.1)	79.2 (11.8)	50/46/40	0.325	0.136	0.677
	BA	79.0 (12.0)	78.1 (10.6)	76.1 (9.5)				
**Caregiver burden**								
**Caregiver burden index** (percentage)	AB	32.4 (14.3)	30.4 (12.1)	24.7 (16.19)	37/36/28	0.548	6.765	**0**.**014**
	BA	29.5 (15.4)	35.2 (15.6)	27.8 (15.6)				

^a^ Higher score indicates better outcome. Values in bold indicate statistically significant results. T1, timepoint 1 (baseline); T2, timepoint 2 (5-month); T3, timepoint 3 (9-month).

In the SIS participation and role function, there was a significant Time main effect over T1–T3 in the AB group [*F*(2,37) = 3.34, *P* = 0.047, η*p*^2^ = 0.129]. *Post hoc* testing showed that outcome improved between T1 and T2 (*P* = 0.016) but not between T1 and T3 (*P* = 0.095) or T2 and T3 (*P* = 0.539), suggesting that the positive effect of the intervention on the social participation of the PWAs was short-term. These comparisons survived FDR-correction. Within the BA group, there was no significant Time main effect [*F*(2,45) = 0.66, *P* = 0.520, η*p*^2^ = 0.019], but the changes were in a positive direction.

In the Caregiver burden index, there were significant within-group T1–T3 changes in both AB [*F*(2,24) = 3.49, *P* = 0.047, η*p*^2^ = 0.226] and BA [*F*(2,34) = 7.07, *P* = 0.003, η*p*^2^ = 0.277] groups. Pairwise comparisons showed that the burden score decreased significantly in the AB group between T1 and T3 (*P* = 0.015), whereas in the BA group it increased between T1 and T2 (*P* = 0.012) and decreased between T2 and T3 (*P* = 0.001), indicating positive effects of the intervention in both groups. These comparisons survived FDR-correction.

### PP analysis

A PP analysis using repeated measures ANOVA was conducted for all the outcome measures that showed significant effects in the ITT analyses (T1 and T2 Time × Group; see above). The PP results were in line with the LMM approach, yielding a Time (T1 and T2)×Group (AB versus BA) interaction that was significant for the Communication index [*F*(1,41) = 5.75, *P* = 0.021, η*p*^2^ = 0.123] and marginally significant for the Responsive speech index [*F*(1,44) = 3.76, *P* = 0.059, η*p*^2^ = 0.079]. Additionally, paired *t*-tests comparing pre- and post-intervention scores (AB: T1 and T2, BA: T2 and T3) across both groups revealed a significant improvement in both the Communication index (*t*_42_ = −3.80, *P* < 0.001) and the Responsive speech index (*t*_41_ = −2.85, *P* = 0.007). In contrast, changes over the control period (AB: T2 and T3, BA: T1 and T2) were not significant (*t*_42_ = −0.16, *P* = 0.874; *t*_43_ = −0.73, *P* = 0.469). Together, these findings support the ITT results on the efficacy of the intervention on communication and spoken language production.

## Discussion

This crossover RCT explored the clinical efficacy of a novel multicomponent singing intervention in chronic aphasia. Our main results between T1 and T2 showed that compared with standard care, the singing intervention (i) enhanced PWAs’ everyday communication ability and spoken language production in tasks involving responsive speech (repetition, naming), (ii) improved PWAs’ social participation and (iii) reduced caregiver burden in FCs. These findings indicate that singing-based rehabilitation, which includes both group- and self-training elements and in which also the FCs can actively participate, can have positive effects on both language functions and psychosocial wellbeing, providing social and emotional support for PWAs and their family members. These findings are clinically important because they provide novel evidence that singing-based interventions coupled with standard care, independent of health care resources, may support recovery of chronic aphasia compared with standard care only.

Most previous studies on group-based singing interventions in aphasia^21–24^ have utilized non-randomized designs or have been feasibility studies and limited by small sample sizes and they have not included an extensive assessment of communication or spoken language production outcomes. The results of the present trial show for the first time that singing-based rehabilitation that includes both group- and self-training elements can improve everyday communication ability (CAL, SIS Communication) and responsive speech (WAB Repetition and Naming) in chronic aphasia. Previously, similar benefits have been observed after intensive MIT or other individual-level singing-based interventions in aphasia.^14–18,40,41^ Our evidence for improvement in communication comes from self- and FC-reports, which are naturally not blinded. However, in aphasia care and research, it is important to include the patient’s own view as an indicator of subjectively experienced outcome,^42^ and FCs can provide valuable complementary information on the everyday functioning and communication of the PWAs, especially in more severe aphasia.^43^ Together with the improved responsive speech observed in standardized tests performed by a blinded investigator, our results capture the broad effects of singing on language function in aphasia. Importantly, the positive effects on both communication ability and responsive speech were maintained 5 months after the cessation of the intervention, which indicates that the verbal benefits induced by the intervention were robust and durable. No significant changes were found in other tasks measuring spontaneous speech, articulatory agility or verbal memory. One potential explanation for the lack of findings in these tasks could be that singing strengthens more automatic phonological language skills, which are linked to left temporoparietal regions in aphasia, while more motor and cognitive elements of connected speech are linked to left frontal regions in aphasia.^44^

Regarding social functioning, the singing intervention showed a positive effect in the SIS participation and role function subscale, in line with previous studies reporting psychosocial benefits of choir singing in healthy seniors^19,20^ and in PWAs.^21^ This effect was short-term and could reflect the increased activity level and the opportunities for engagement, social interaction, and peer support experienced by the PWAs in an enriched communicative environment.^45^ No effects were observed on the PWAs’ self-reported mood (CES-D) or social support (SPS) or in more generic functional outcome (other SIS scales), which is somewhat surprising, because music-based interventions have previously been linked to mood and QoL benefits in healthy^19,20^ and neurological^9–11^ populations. This may reflect the difficulty of questionnaire-based measurement of subjective emotional wellbeing in aphasic patients and the need for a larger sample size to detect effects.^24^

Finally, we observed a long-term reduction in caregiver burden following the singing intervention, in line with similar findings of FCs in dementia.^46^ This may be related to the positive self-experienced emotional impact of choir singing,^19,20^ the increased interaction with the PWA and other FCs (including peer support) during the intervention, or to the intervention-induced communicative and psychosocial improvements of the PWA. This finding is important given the high prevalence of mood disorders in the FCs of PWAs.^8^

Regarding the commitment and adherence of the patients to the intervention protocol, the attendance rates for group training were high (around 90%), whereas the amount of home training was more variable. Originally, the patients were instructed to have three 30 min home training sessions each week (total 24 h over 16 weeks), but the realized total amount of home training was markedly lower (on average 11.9 h of using the Singalonger; however, there was a lot of individual variability, with some patients training almost 40 h with the app). There are likely a number of factors contributing to this variability (e.g. motivation, cognitive problems, attitudes, technical issues), which will be separately analysed and reported along with other intervention and usability feedback as well as dosage issues, important for the applicability of the intervention model.

The present study has following potential limitations. First, while being the largest study to date on group-based singing in aphasia, our sample is moderate in size and comprises PWAs of varying severity; in future, large-scale studies are warranted to determine the efficacy of singing across different aphasia types and severity levels. Second, while there was a statistically significant improvement in everyday communication abilities with a large effect size, the direct clinical relevance of this change is not known as there are no standardized estimates for minimal clinically important difference for CAL. Third, although the multicomponent nature of the intervention likely contributes to its broad efficacy in both communicative and psychosocial domains, it also precludes making inferences about the contribution of each component (group-based singing, group-MIT, tablet-based home training) on the outcomes. Fourth, the crossover design bears some methodological considerations: due to the possible carry-over effect in the AB group between T2 and T3, intervention effects in the BA group could not be reliably estimated (compared with standard care). To gain a more comprehensive understanding of the changes over the entire follow-up period (T1–T3), we performed within-group analysis in both intervention groups. Whereas the AB group showed significant findings consistent with our hypothesis between T1 and T2 (with treatment-induced gains in communication, responsive speech and caregiver burden maintained also longitudinally up to T3), the changes in the BA group between T2 and T3 were in a positive direction, but did not reach statistical significance, with the exception of caregiver burden. However, a pooled analysis of the AB and BA groups showed significant improvement in communication and responsive speech over the intervention period whereas there were no significant changes during the control period. It is possible that motivational factors play some role, as the BA group had to wait 5 months (and undergo two assessment points) before receiving the intervention. Furthermore, while the groups did not show significant differences in clinical or biographical background information associated with therapy response in chronic aphasia,^47^ there might be specific biographical, neuropsychological, or neurobiological factors influencing treatment response to singing-based interventions in chronic aphasia that have remained yet uncharted. Future multimodal studies exploring the predictors of therapy response to singing interventions are needed and would help clinicians to individualize treatment strategies to optimize recovery.

Despite these limitations, the observed effects are encouraging in suggesting that singing may be a potential tool to promote communicative and psychosocial outcome even in chronic post-stroke aphasia as well as provide a meaningful joint activity for PWAs and FCs that can also alleviate the burden experienced by the caregivers. Importantly, these positive findings (i) provide further support for recent evidence that rehabilitation interventions can achieve significant improvements in core outcomes, such as motor, cognitive or verbal abilities, still in the chronic stage, years after stroke^5,6,48^ and that (ii) singing-based interventions can be a powerful tool to unlock communicative skills in chronic aphasia, possibly mediated by the largely bilateral engagement of vocal-motor and auditory brain regions associated with singing.^13,49–51^ Notably, the multicomponent singing intervention used in the present study included elements of choir singing, singing-based speech training, tablet-assisted home training, and PWA-FC interaction and was implemented by two professionals with expertise on music therapy in neurological patients and on choir conduction and singing instruction. Our results demonstrate this type of novel intervention model provides a versatile, motivating, scalable and potentially cost-effective approach to aphasia rehabilitation.

## Data Availability

Anonymized data reported in this manuscript are available from the corresponding author upon reasonable request and subject to approval by the appropriate regulatory committees and officials.

## Abbreviations

ADL =: activities of daily living
BDAE =: Boston Diagnostic Aphasia Examination
CAL =: Communicative Activity Log
CES-D =: Center for Epidemiological Studies—Depression
CONSORT =: Consolidated Standards of Reporting Trials
FC =: family caregiver
FDR =: false discovery rate
GHQ-12 =: General Health Questionnaire
IADL =: instrumental activities of daily life
ITT =: intention-to-treat
LMM =: linear mixed effects model
MIT =: melodic intonation therapy
PP =: per-protocol
PWA =: person with aphasia
QoL =: quality of life
RCT =: randomized controlled trial
SIS =: Stroke Impact Scale
SPS =: Social Provision Scale
TIDieR =: Template for Intervention Description and Replication
T1 =: timepoint 1 (baseline)
T2 =: timepoint 2 (5-month)
T3 =: timepoint 3 (9-month)
WAB =: Western Aphasia Battery
ZBI-22 =: Zarit Burden Interview
